# Artificial rearing with milk replacers of varying fat and total solids compared with natural suckling: Pre- and post-weaning performance of lambs

**DOI:** 10.1016/j.vas.2026.100839

**Published:** 2026-08-27

**Authors:** Ameneh Akbarizadeh, Ebrahim Ghasemi, Meysam Gholami, Bahareh Dolatkhah

**Affiliations:** a Department of Animal Science, College of Agriculture, Isfahan University of Technology, Isfahan, 84156-83111, Iran; b Novin Roshd Shahran Fodeh Company, Research and Development Department, Isfahan, Iran

**Keywords:** Milk replacer, Artificial rearing, Total solids, Milk fat, Preweaning growth

## Abstract

This study evaluated the effects of increasing total solids (TS) and fat concentration in milk replacer (MR) on the performance of artificially reared lambs compared with naturally suckled lambs. Forty-eight 3-day-old Shal lambs were assigned to natural suckling (EWE; 6.37% fat, 17.4% TS) or one of three MR treatments: low-fat–low-solids (LF-LS; 3.56% fat, 16.3% TS), low-fat–high-solids (LF-HS; 4.27% fat, 19.6% TS), or high-fat–high-solids (HF-HS; 4.95% fat, 19.6% TS). Increasing TS raised the milk protein concentration to a level comparable to that of ewe milk (4.8%), while partial replacement of lactose with fat reduced osmolality in HF-HS relative to LF-HS (466 vs. 506 mOsm/kg). Lambs were fed milk until day 62 and monitored until day 103. Lambs fed LF-HS and HF-HS consumed more protein and metabolizable energy than those fed LF-LS, resulting in improved pre- and postweaning average daily gain and skeletal growth. Furthermore, LF-LS lambs exhibited lower serum cholesterol, a tendency toward lower glucose, and higher β-hydroxybutyrate concentrations at weaning, indicating a less favorable energy status. Although HF-HS increased nutrient intake and hip width compared with LF-HS, both high-solids formulations achieved growth comparable to that of EWE. Nutrient digestibility, diarrhea days, and serum concentrations of insulin, cortisol, and thyroxine were unaffected, whereas serum total protein was greater in EWE than in the MR groups. Despite the associated increase in osmolality, increasing MR solids from 16.3% to 19.6%, particularly in HF-HS, improved nutrient intake and supported growth comparable to natural suckling without adversely affecting the assessed health indicators.

## 1 Introduction

Optimizing neonatal nutrition is essential for improving survival, health, and growth in modern intensive and semi-intensive sheep production systems. This challenge is particularly important in meat-type flocks, where increasing litter size often exceeds the milk-producing capacity of ewes, and in dairy sheep systems, where milk is diverted for commercial sale. Consequently, artificial rearing using milk replacer (MR) has become an important nutritional strategy to improve lamb survival and support early growth.

Adequate nutrition during the preweaning period is a key determinant of growth, body composition, immune competence, health, and survival in young ruminants (Amdi et al., 2021; Battacone et al., 2024; Geiger et al., 2016; Hu et al., 2020; Mahjoubi et al., 2017; Zhang et al., 2019). Because this stage is characterized by rapid tissue accretion and organ development, nutritional deficiencies during early life can have lasting consequences that are not fully compensated after weaning (Bhatt et al., 2009). Consequently, optimizing nutrient supply during the milk-feeding period has become an important objective in modern sheep production systems, particularly where artificial rearing is used to improve lamb survival and increase the availability of marketable ewe milk. Unlike naturally suckled lambs, artificial rearing replaces ewe milk with MR and eliminates maternal nursing, making the nutritional quality of the replacer a critical determinant of animal performance. In such systems, nutrient supply can be increased by offering larger volumes of MR, increasing its total solids (TS) concentration, or modifying its nutrient composition. Although increasing milk allowance generally enhances preweaning growth, it often reduces starter intake, delays rumen development, and may compromise postweaning performance (Huang et al., 2023). Increasing the TS concentration, rather than feeding volume, has therefore attracted considerable interest because it increases nutrient intake while having minimal effects on passage rate, starter intake, nutrient digestibility, and rumen development (Azevedo et al., 2023). Similarly, optimizing the protein-to-energy ratio and increasing dietary fat concentration have been reported to improve growth performance and feed efficiency in young ruminants (Fukami et al., 2025; Herath et al., 2020).

Most research on MR formulation has been conducted in dairy calves, although MR developed for calves are also widely used for artificial lamb rearing (Mahjoubi et al., 2017; Mousavi Esfiokhi et al., 2024). However, ewe milk contains greater concentrations of TS (16–18% vs. 12–13%), fat (5.5–7% vs. 3–4%), and protein (4.5–6% vs. 3–3.5%), whereas lactose accounts for a smaller proportion of dry matter (approximately 30% vs. 40%) (NRC, 2001, 2007). Despite these compositional differences, the osmolality of ewe and cow milk is similar (approximately 280–302 mOsm/kg). These characteristics suggest that MR formulations for lambs should more closely resemble ewe milk in nutrient density without substantially increasing osmotic load. Increasing the TS concentration of MR is an effective strategy to increase nutrient intake and promote preweaning growth (Shiasi Sardoabi et al., 2021). Nevertheless, increasing TS by adding lactose also increases osmolality, which may impair abomasal emptying and increase the risk of digestive disorders (Azevedo et al., 2016). Partial replacement of lactose with fat offers a practical alternative because it increases dietary energy density with only a limited effect on osmolality (Litherland et al., 2014).

Despite these advances, important knowledge gaps remain. Previous studies have mainly examined individual nutritional factors, such as milk allowance, protein concentration, or dietary fat, whereas optimization of TS in MR has received comparatively little attention in lambs. Moreover, few studies have evaluated high-solids MR formulations specifically designed to resemble ewe milk or directly compared them with natural suckling under identical management conditions. Consequently, it remains unclear whether increasing MR TS while partially replacing lactose with fat can improve growth without compromising digestive function or postweaning performance.

We hypothesized that increasing MR TS while maintaining protein and metabolizable energy concentrations comparable to ewe milk, together with partial replacement of lactose by fat to control osmolality, would improve growth performance in artificially reared lambs. Therefore, the objective of this study was to compare natural suckling with artificial rearing using MR formulations differing in TS and fat concentrations and to evaluate their effects on growth performance, digestibility, metabolic parameters, and selected health indicators (based on clinical scoring) in neonatal lambs.

## 2 Material and methods

### 2.1 Animals, treatments, and management

The experiment was conducted at the Lavark Educational and Research Farm, College of Agriculture, Isfahan University of Technology (Isfahan, Iran). Forty-eight neonatal Shal lambs (24 males and 24 females), born within a 10-d period, were enrolled in the study. Immediately after birth, lambs were dried by their dams, weighed, and their navels were disinfected with 7% iodine tincture. To ensure adequate passive transfer of immunity, lambs remained with their dams and had free access to colostrum during the first 48 h after birth.

**Fig. 1:**
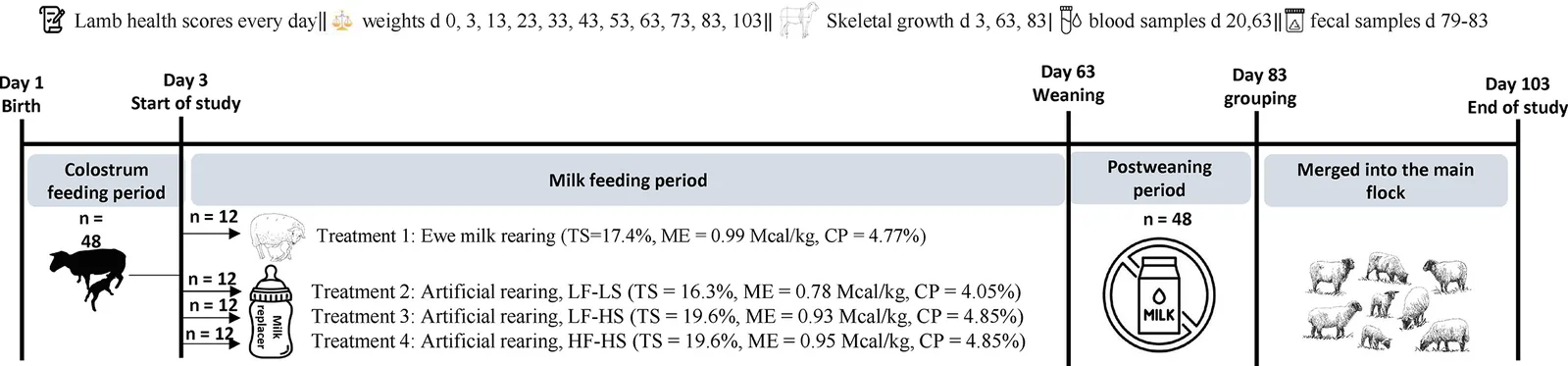
Schematic representation of the experimental timeline and design. Lambs received colostrum from dams during days 0–2. On day 3, lambs were weighed and randomly assigned to four treatments: (1) Ewe milk, (2) LF-LS, (3) LF-HS, and (4) HF-HS. Weaning occurred on day 63. During the postweaning period (from 63 to 83 days), lambs remained in their pens. Thereafter, lambs were merged into the main flock and monitored until day 103.

On day 3, lambs were weighed and randomly allocated to one of four treatments: (1) natural rearing with ewe milk (EWE); (2) low-fat–low-solids MR (LF-LS); (3) low-fat–high-solids MR (LF-HS); or (4) high-fat–high-solids MR (HF-HS). Each treatment included 12 lambs, with an equal number of males and females (6 males and 6 females). Moreover, each treatment included 8 single-born lambs and 4 twin-born lambs. For twin-born lambs, only one lamb from each twin-bearing ewe was enrolled per treatment. Allocation based on birth date ensured comparable mean age among treatments at the beginning of the experiment. The MR treatments were prepared from two commercial MR powders (Novin Roshd Shahran Fodeh Co., Isfahan, Iran). Both products contained 25% crude protein (CP; DM basis) but differed in fat concentration, providing either 22.5% (low-fat) or 25.4% fat (high-fat) on a DM basis (Table 1). The formulations consisted of skim milk powder, whey protein concentrate, whey powder, vegetable fat (palm oil), minerals, vitamins, and feed additives. The LF-LS MR was prepared by mixing 1 kg of low-fat powder with 5 L of water, producing a solution containing 16.3% total solids (TS). The LF-HS and HF-HS MRs were prepared by mixing 1 kg of the corresponding powder with 4 L of water, resulting in a TS concentration of 19.6%. MRs were prepared fresh each day using water at 45–50 °C and were offered at 38–40 °C in 1.5-L feeding bottles. The experimental design and sampling schedule are presented in Fig. 1.

**Table 1:** Ingredient and nutrients composition (DM basis) of ewe milk, milk replacer with low and high fat and starter diet used in this experiment.

Item	Ewe milk	Milk replacer with low fat^a^	Milk replacer with high fat^a^	Starter diet^b^
DM, % as-fed	17.4	97.0	97.0	89.5
Fat, %	36.7	22.5	25.4	2.30
CP, %	27.5	25.0	25.0	17.5
NDF, %	-	-	-	19.9
Starch, %	-	-	-	43.7
Total sugars, %^c^	30.7	46.6	42.9	4.0
Ash, %	5.2	6.4	7.1	7.7
ME, Mcal/kg	5.69	4.80	4.92	2.95

a Hero Milk Replacer, Novin Roshdeh Shahran Fodeh Company (Isfahan, Iran).

b Containing (DM basis): 10% alfalfa hay, 57.9% corn grain, 23.8% soybean meal, 4.5% wheat bran, 1.2% CaCo3, 0.18% MgO, 0.9% NaHCO3, 0.3% NaCl, 0.9% vitamin premix (1300,000 IU of vitamin A; 360,000 IU of vitamin D3; 12,000 IU of vitamin E), 0.36% mineral premix (0.8 g of iron; 16 g of zinc; 10 g of manganese; 4 g of copper; 0.15 g of iodine; 0.12 g of cobalt; and 0.08 g of selenium).

c Disaccharides + monosaccharides.

**Fig. 2:**
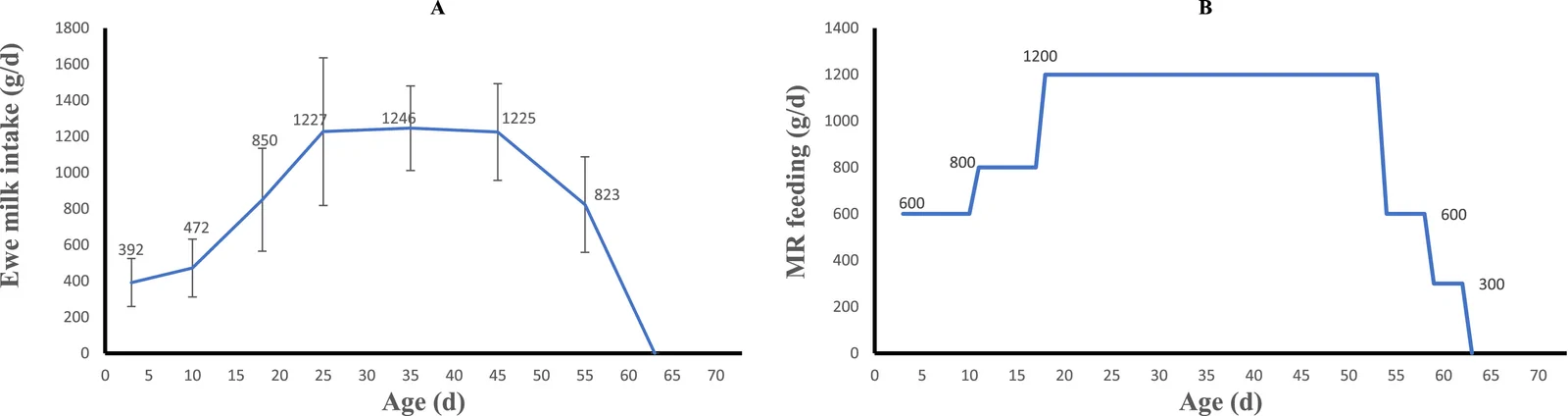
Fresh milk intake in lambs on ewe milk (A) or different MR (B). Data for lambs on ewe milk were expressed as means ± SD.

Lambs assigned to the EWE treatment suckled their dams twice daily (09:00–09:30 and 16:00–16:30 h) from day 3 to 53 and once daily (16:00–16:30 h) from day 54 until weaning (day 62). Lambs assigned to the MR treatments were artificially reared according to a fixed feeding schedule: 600 mL/d from day 3 to 10, 800 mL/d from day 11 to 17, 1200 mL/d from day 18 to 53, and 600 mL/d from day 54 to 58, divided equally between the morning and afternoon feedings. From day 59 to 62, lambs received 300 mL/d in the morning only as part of the weaning program (Fig. 2). Lambs were housed in groups of four in pens measuring 2.0 × 1.2 m, providing approximately 0.6 m² per lamb, with three replicate pens per treatment. Each pen was equipped with a feed bunk (0.35 × 1.0 × 0.15 m) and a water bucket (0.15 × 0.80 × 0.25 m). Starter concentrate (Table 1) and fresh water were available ad libitum throughout the study. Salt blocks were introduced on day 20, and straw bedding was replenished as required to maintain a clean and dry environment. All lambs were weaned on day 63 and remained in their original pens until day 83 for continued monitoring. Thereafter, lambs were transferred to a common flock and monitored until day 103.

### 2.2 Measurements

Lambs were weighed individually at birth, on day 3, every 10 days thereafter until day 83, with all measurements performed at 10:00 h. An additional body weight was recorded on day 103 to evaluate postweaning growth. Skeletal growth measurements, including wither height (highest point of the withers), hip height (distance from the tuber coxae to the ground), hip width (distance between the left and right tuber coxae), body length (distance from the last cervical vertebra to the tuber coxae), and chest circumference (measured immediately caudal to the shoulders), were obtained on days 3, 63, and 83.

Milk intake of ewe-reared lambs (EWE) was estimated using the weigh–suckle–weigh method. Lambs were weighed immediately before and after each 30-min suckling period (09:00–09:30 and 16:00–16:30 h) using a digital scale with an accuracy of ±10 g. The difference between pre- and post-suckling body weight was considered the milk consumed during each suckling bout. Milk composition, including fat, protein, lactose, and total solids, was determined using a MilkoScan milk analyzer (Foss Analytical A/S, Hillerød, Denmark) according to the manufacturer's instructions. Osmolality of the reconstituted MR solutions was measured by freezing-point depression using an OSMOMAT 3000 osmometer (Gonotec GmbH, Berlin, Germany).

Lamb health was evaluated daily before the morning feeding by two trained observers throughout the experimental period. Clinical assessments included fecal consistency, general appearance, respiratory status, and ocular discharge. These health indicators were scored according to the standardized clinical scoring systems for ruminants described by McGuirk (2008). Briefly, fecal consistency and respiratory health (cough score) were evaluated on a 5-point scale (1 = normal to 5 = severe), while general appearance and ocular discharge were assessed on a 4- to 5-point scale based on the severity of clinical signs. For each lamb, the number of days with abnormal scores was recorded. Clinical observations and diagnoses were performed daily using standardized, reproducible criteria. Diarrhea was diagnosed when a lamb presented with a fecal consistency≥3. Pneumonia (respiratory disease) was diagnosed based on the McGuirk health scoring system, defined by the presence of a cough score≥2 accompanied by a rectal temperature>40 °C. All clinical observations and diagnoses were verified by the station veterinarian.

Fecal samples for determination of apparent nutrient digestibility were collected individually by gentle rectal stimulation each morning (09:00–11:00 h) for five consecutive days during the postweaning period (days 79–83). Samples collected from each lamb were pooled, dried at 60 °C for 48 h, ground to pass a 1-mm screen, and stored at −20 °C until analysis. Apparent nutrient digestibility was determined using acid-insoluble ash (AIA) as an internal marker according to the procedure of Van Keulen and Young (1977). Briefly, feed and fecal samples were ashed at 550 °C for 6 h, and the residue was boiled with 2 N HCl for 5 min. The insoluble residue was filtered, washed, and re-ashed. AIA concentration was determined gravimetrically.

Blood samples were collected from all lambs via jugular venipuncture between 12:00 and 13:00 h on days 20 and 63 (weaning) using evacuated blood collection tubes. Serum was separated within 2 h after collection by centrifugation at 3000 × g for 15 min at 4 °C and stored at −20 °C until analysis. Serum concentrations of glucose (enzymatic method using glucose oxidase-phenol 4-aminoantipyrine peroxidase, 505 nm; Catalog number: 117,500), cholesterol (enzymatic method using cholesterol oxidase-phenol 4-aminoantipyrine peroxidase, 546 nm; Catalog number: 110,500), urea (enzymeatic method using urease-GLDH, Catalog number: 141,500), albumin (photometric test using bromocresol green, 546 nm; Catalog number: 101,500), and total protein (Biuret method, 560 nm; Catalog number: 128,500) were determined using commercial assay kits (Pars Azmoon, Tehran, Iran) with an automated biochemical analyzer (Hitachi 902, Roche Diagnostics, Mannheim, Germany). Serum concentrations of thyroxine (T4), cortisol, and insulin were measured using commercial enzyme-linked immunosorbent assay (ELISA) kits (Monobind Inc., Lake Forest, CA, USA; catalog numbers: T4 225–300, Cortisol 2725–300, and Insulin 2775–300) according to the manufacturer's instructions. The intra- and inter-assay coefficients of variation for these analytes were <5% and <9.5%, respectively. Serum β-hydroxybutyrate (BHB) concentration was determined using a commercial assay kit (β-hydroxybutyrate dehydrogenase method, 340 nm, Randox Laboratories Ltd., Ardmore, UK), with intra- and inter-assay coefficients of variation of <4.5% and <2.9%, respectively.

### 2.3 Calculation and statistical analysis

Apparent digestibility (%) of DM was calculated using the following equation:

Digestibility(%)=100−[100×(AIA_feed/AIA_feces)]

Gross energy (GE; kcal/kg) of ewe milk and MRs was estimated using the equation:

GE=(0.057×CP)+(0.092×fat)+(0.0395×lactose),

where CP, fat, and lactose are expressed as percentages on a DM basis. Content of ME was estimated as 0.93 × GE for ewe milk and 0.91 × GE for MR according to the conversion factors described by NRC (2001).

Performance data were analyzed over three phases: preweaning (days 3–63), postweaning (days 64–83), and overall experimental period (days 3–83). The pen (n = 3 per treatment) was the experimental unit for starter intake, whereas individual lambs (n = 12 per treatment) were considered the experimental unit for all other variables. Pen nested within treatment was included as a random effect to account for within-pen correlation. Dam and litter effects were partially confounded with the rearing system and thus could not be modeled independently. An a priori power analysis confirmed that 12 lambs per treatment provided 80% power (α = 0.05) to detect a 30 g/day difference in preweaning ADG.

Repeated-measures variables (BW, skeletal measurements) were analyzed using PROC MIXED (SAS 9.4) including treatment, time, birth type, and their interaction as fixed effects. Day 3 measurements were included as covariates to adjust for baseline differences. Covariance structures (CS, CSH, AR(1), heterogeneous AR(1), UN) were compared; AR(1) provided the best fit (lowest AIC) for all repeated measures. Degrees of freedom were estimated using the Kenward–Roger approximation. Model assumptions were evaluated by inspection of residual plots. The statistical model was:

$$Y=\mu +T+D+S+B+\left(T\times D\right)+\beta \left(X-X^{-}\right)+P\left(T\right)+\varepsilon ,$$

where Y = dependent variable, μ = overall mean, T = treatment, D = time, S = sex, B = birth type, T × D = interaction, β(X−X̄) = baseline covariate adjustment, P(T) = random effect of pen nested within treatment, and ε = residual error. Single-measure variables were analyzed using ANCOVA with baseline day 3 values as covariates.

Health outcomes were analyzed using PROC GLIMMIX with appropriate distributions: multinomial for ordinal scores and Poisson for diarrhea days. For GLIMMIX models, the Laplace estimation method was used, and residual plots were examined to confirm model fit. Three pre-planned orthogonal contrasts were tested: (1) EWE vs. all MR (T1 vs. T2–T4); (2) low-solids vs. high-solids MR (T2 vs. T3+T4); and (3) low-fat vs. high-fat at high solids (T3 vs. T4). No additional multiplicity adjustment was applied due to the orthogonal and pre-planned nature of these contrasts. Results are presented as least square means ± SEM. Significance was declared at P ≤ 0.05, with tendencies considered at 0.05 < P ≤ 0.10.

## 3 Results

### 3.1 Composition and intake of milk and starter

Compared with ewe milk, all MR formulations contained lower fat concentrations but substantially greater total sugars, both on a DM basis (Table 1) and in the reconstituted form (Table 2). The protein concentration in the high-solids MR treatments (LF-HS and HF-HS) was comparable to that of ewe milk, whereas LF-LS contained less protein. Notably, the HF-HS treatment provided CP and ME concentrations similar to those of ewe milk (4.85%vs. 4.77% CP and 0.95 vs. 0.99 Mcal/kg ME). The osmolality of all MR formulations exceeded the physiological range of ewe milk (298 mmol/kg; Table 2). The highest osmolality was observed in LF-HS (506 mmol/kg), whereas the lowest was in LF-LS (423 mmol/kg). HF-HS showed an intermediate osmolality (466 mmol/kg).

**Table 2:** Fat, protein, lactose, total solids, density, and metabolizable energy of fresh ewe milk and reconstituted milk replacer (MR) with low fat-low total solids (LF-LS), low fat-high total solids (LF-HS), and high fat-high total solids (HF-HS).

	Ewe milk	LF-LS	LF-HS	HF-HS
Total solids, %	17.4	16.3	19.6	19.6
Fat, %	6.37	3.56	4.27	4.95
CP, %	4.77	4.05	4.85	4.85
Total sugars^a^, %	5.33	7.55	9.04	8.32
Ash, %	0.90	1.04	1.24	1.38
GE, Mcal/kg	1.07	0.86	1.03	1.06
ME, Mcal/kg	0.99	0.78	0.93	0.95
CP:ME, g/Mcal	48.1	51.9	52.1	51.0
CP: Fat, g/g	0.75	1.14	1.13	0.98
Osmolality, mOsm/kg	298	423	506	466
Density, g/ml	1.19	1.01	1.02	1.02

a Lactose + monosaccharides.

**Fig. 3:**
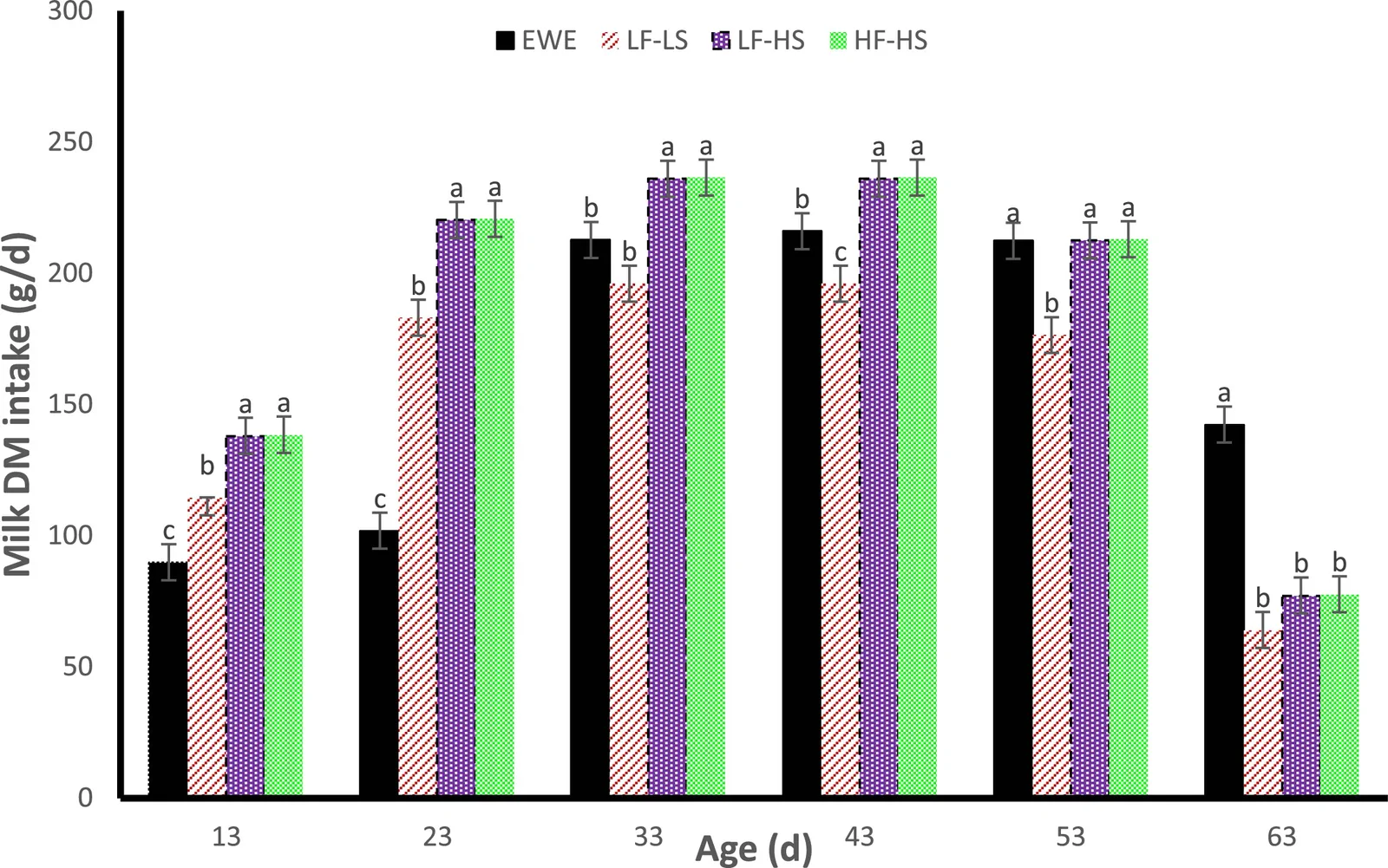
Milk DM intake of lambs across experimental treatments (EWE, LF-LS, LF-HS, and HF-HS). Data are presented as means ± SEM. Means with different superscripts (a, b, c) differ significantly (P < 0.05).

**Fig. 4:**
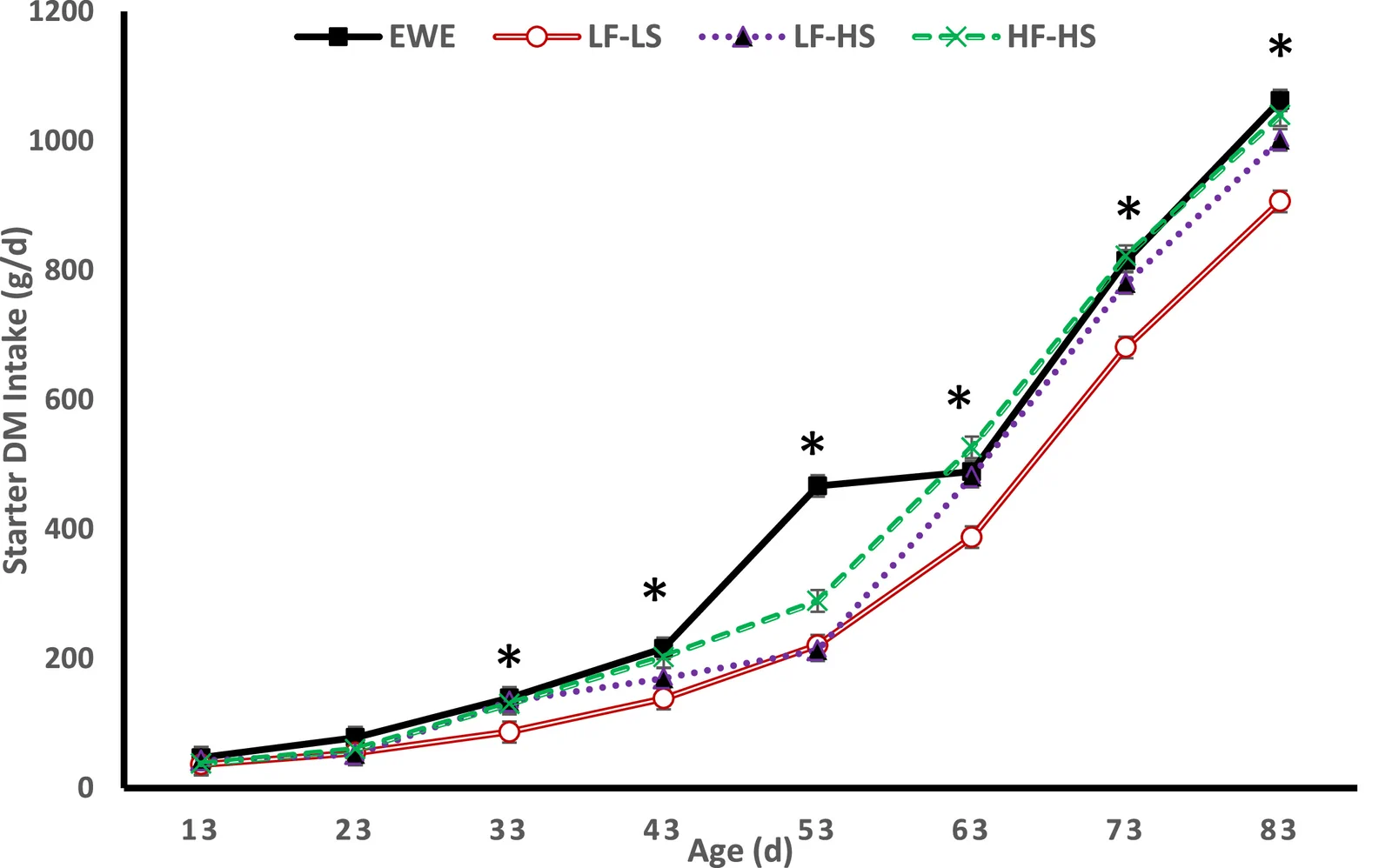
Starter DM intake of lambs from day 3 to 83 across experimental treatments (EWE, LF-LS, LF-HS, and HF-HS). Data are presented as means ± SEM. * P < 0.05.

Over the preweaning period (days 3–63; Fig. 3), lambs fed LF-LS consumed the same amount of milk DM as EWE lambs (155 g/d), but significantly less than lambs fed LF-HS (187 g/d) or HF-HS (188 g/d; Table 3). In contrast, starter DM intake was highest in EWE (234 g/d), lowest in LF-LS (154 g/d), and intermediate in LF-HS (182 g/d) and HF-HS (208 g/d). Compared with LF-HS, HF-HS lambs consumed more starter DM during the preweaning period and had greater total ME intake during both the preweaning and overall periods. Although starter intake was low during the first 3 weeks across all groups, differences among treatments became evident from day 23 onward and persisted until day 83 (Fig. 4). Over the entire experimental period (days 3–83), total CP intake was lowest in LF-LS (84.0 g/d) and greatest in EWE (104 g/d) and HF-HS (103 g/d), with LF-HS being intermediate (97.9 g/d; Table 3). Total ME intake was lowest in LF-LS (1.48 Mcal/d) and greatest in EWE (1.87 Mcal/d), with HF-HS exceeding LF-HS (1.84 vs. 1.73 Mcal/d). Similarly, total DM intake (milk + starter) was consistently lowest in LF-LS (430 g/d) and greatest in EWE (527 g/d) and HF-HS (529 g/d), with LF-HS tending to be lower than HF-HS (499 g/d; P = 0.07).

**Table 3:** Milk (n = 12 per treatment) and starter DM intake (n = 3 pens per treatment), ADG and feed efficiency in lambs (n = 12 per treatment) on natural rearing with ewe milk (EWE) or MR with low fat-low total solids (LF-LS), low fat-high total solids (LF-HS), and high fat-high total solids (HF-HS).

	Treatment					*P*-value^a^		
Item		MR				EWE vs.	Low vs.	Low vs.
	EWE	LF-LS	LF-HS	HF-HS	SEM	MR	high solid	high fat
Milk intake								
DM, g/d	155	155	187	188	3.85	<0.01	<0.01	0.91
ME, Mcal/d	0.881	0.746	0.900	0.927	0.021	0.36	<0.01	0.38
Starter intake, g/d								
3–63 d	234	154	182	208	8.19	<0.01	<0.01	0.03
63–83 d	940	793	890	929	22.2	0.01	<0.01	0.21
3–83 d	411	314	359	388	10.8	<0.01	<0.01	0.06
Total DMI, g/d								
3–63 d	389	309	370	396	9.23	<0.01	<0.01	0.05
63–83 d	940	793	890	929	22.2	0.01	<0.01	0.21
3–83 d	527	430	499	529	11.3	<0.01	<0.01	0.07
Total ME intake, Mcal/d								
3–63 d	1.57	1.20	1.44	1.54	0.033	<0.01	<0.01	0.03
63–83 d	2.77	2.34	2.62	2.74	0.065	0.01	<0.01	0.21
3–83 d	1.87	1.48	1.73	1.84	0.036	<0.01	<0.01	0.04
Total CP intake, g/d								
3–63 d	83.6	65.8	78.7	83.4	1.80	<0.01	<0.01	0.07
63–83 d	151	120	138	145	3.60	<0.01	<0.01	0.16
3–83 d	104	84.0	97.9	103	2.05	<0.01	<0.01	0.08
ADG, g/d								
3–63 d	221	180	224	237	10.19	0.54	<0.01	0.38
63–83 d	306	264	281	298	15.6	0.19	0.20	0.46
3–83 d	242	202	239	252	9.61	0.32	<0.01	0.34
Feed efficiency, g of ADG to g of total DMI								
3–63 d	0.568	0.582	0.615	0.565	0.028	0.57	0.83	0.22
63–83 d	0.325	0.337	0.312	0.325	0.018	0.96	0.41	0.63
3–83 d	0.459	0.469	0.477	0.476	0.021	0.52	0.75	0.98
Nutrient digestibility, %								
DM	77.8	78.9	78.0	79.1	1.06	0.50	0.78	0.44

a EWE vs. MR: T1 (EWE) vs. T2+T3+T4 (all MR treatments), Low vs. high solid: T2 (LF-LS) vs. T3+T4 (LF-HS + HF-HS), Low vs. high fat: T3 (LF-HS) vs. T4 (HF-HS).

### 3.2 Growth and body measurements

**Fig. 5:**
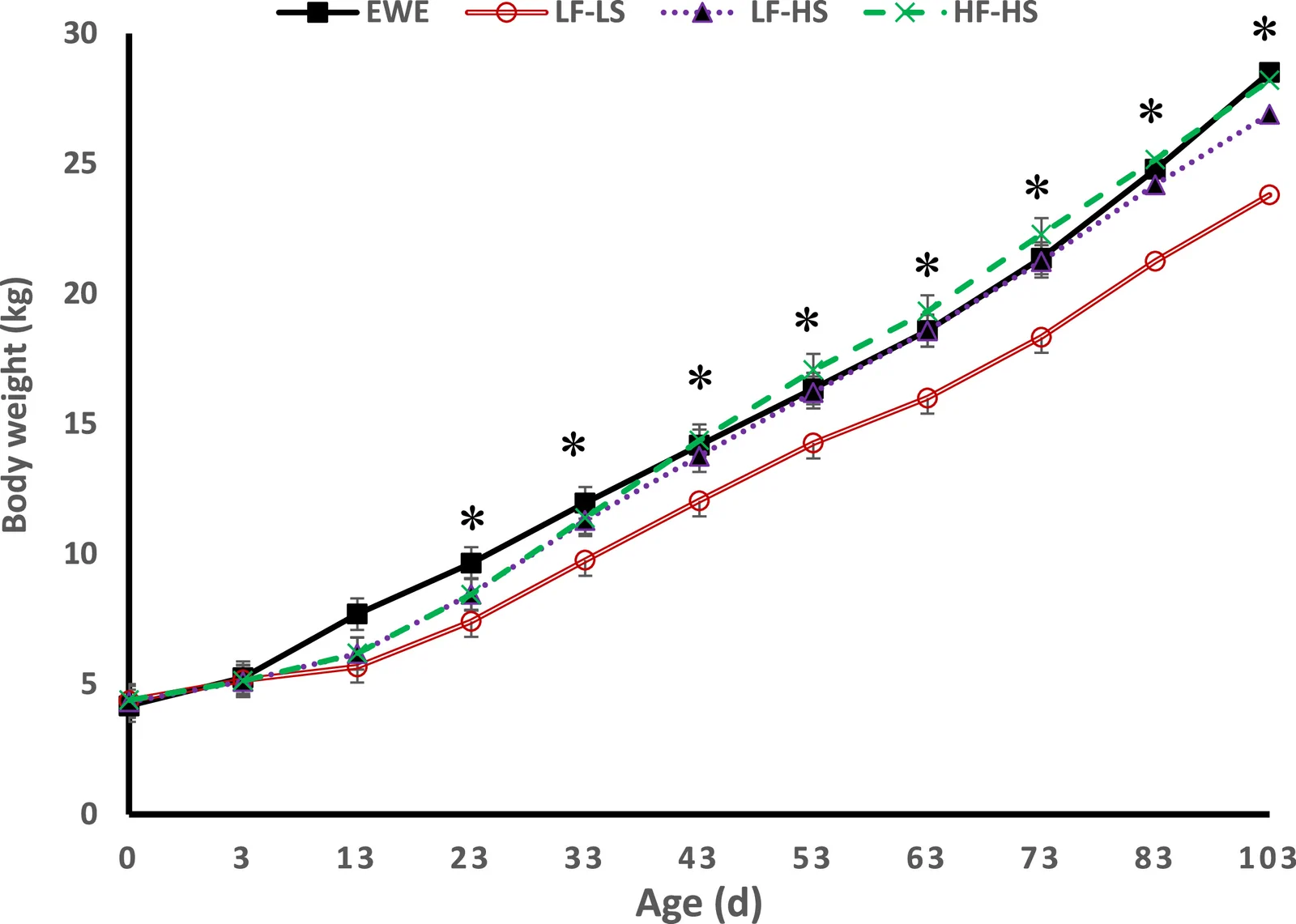
Body weight of lambs from day 0 to 103 across experimental treatments (EWE, LF-LS, LF-HS, and HF-HS). Data are presented as means ± SEM. * P < 0.05.

Body weight differed significantly among treatments, primarily during the milk-feeding period (Fig. 5). Lambs fed LF-LS consistently weighed less than those in all other groups from early in the trial, and this difference increased until weaning (d 63). In contrast, lambs on EWE, LF-HS, or HF-HS exhibited similar BW throughout the study. Although all groups gained weight during the postweaning period (d 63–103), LF-LS lambs did not fully compensate for their early growth restriction and had lower final BW on d 103. No differences in BW were detected among EWE, LF-HS, and HF-HS lambs at any measurement time.

These patterns were reflected in ADG during the preweaning period (d 3–63): lambs on EWE gained more than those fed LF-LS (221 vs. 180 g/d), but ADG did not differ between EWE and either LF-HS (224 g/d) or HF-HS (237 g/d; Table 3). No treatment differences in ADG were observed during the postweaning (d 63–83) or overall (d 3–83) periods, and ADG was similar between LF-HS and HF-HS across all phases. Feed efficiency (ADG per unit of total DMI) was not affected by treatment during all measured periods (Table 3). Similarly, DM digestibility did not differ among treatments and ranged from 77.8% to 79.1%.

At weaning (d 63), hip height was greater in lambs on HF-HS and LF-HS than in those on LF-LS (Table 4). Withers height was also greater in HF-HS than EWE (55.0 vs. 52.7 cm). By d 83, withers height remained greater in both HF-HS and LF-HS than in LF-LS but was similar to that of EWE. Hip width at day 83 was greater in HF-HS than in LF-HS lambs, whereas body barrel circumference did not differ among treatments at any time point.

**Table 4:** Body measurements in lambs (n = 12 per treatment) on natural rearing with ewe milk (EWE) or MR with low fat-low total solids (LF-LS), low fat-high total solids (LF-HS), and high fat-high total solids (HF-HS).

Item	Treatment					*P*-value^a^		
		MR			SEM	EWE vs.	Low vs.	Low vs.
	EWE	LF-LS	LF-HS	HF-HS		MR	high solid	high fat
Body weight, kg								
d3	5.25	5.33	5.25	5.28	0.17	0.84	0.75	0.89
d63	18.7	15.9	18.5	19.1	0.65	0.25	<0.01	0.49
d83	24.9	21.2	24.0	25.0	0.83	0.12	<0.01	0.44
d103	28.6	23.7	26.8	28.0	1.08	0.05	<0.01	0.43
Body barrel, cm								
d3	49.0	47.7	49.8	49.5	1.0	0.97	0.14	0.80
d63	61.7	60.6	62.8	63.0	0.79	0.60	0.03	0.82
d83	67.4	63.7	66.2	66.7	0.79	0.05	<0.01	0.65
Body length, cm								
d3	42.2	41.4	43.0	42.0	0.86	0.93	0.28	0.43
d63	53.3	51.5	53.1	55.1	0.72	0.89	<0.01	0.05
d83	57.5	55.3	56.2	56.5	1.33	0.36	0.52	0.87
Hip height, cm								
d3	44.7	44.6	46.8	46.1	0.79	0.23	0.05	0.52
d63	54.7	54.2	56.0	56.6	0.53	0.14	<0.01	0.38
d83	58.7	56.8	58.8	60.2	0.68	0.85	<0.01	0.16
Withers height, cm								
d3	44.3	43.2	45.8	44.8	0.76	0.68	0.03	0.37
d63	52.7	53.0	54.5	55.0	0.56	0.03	0.20	0.53
d83	56.4	55.5	57.6	58.1	0.70	0.42	0.01	0.56
Hip width, cm								
d3	10.5	9.87	10.1	10.0	0.30	0.20	0.58	0.83
d63	13.0	12.9	13.1	13.3	0.25	0.70	0.36	0.62
d83	14.3	14.0	14.1	14.8	0.26	0.94	0.17	0.04

a EWE vs. MR: T1 (EWE) vs. T2+T3+T4 (all MR treatments), Low vs. high solid: T2 (LF-LS) vs. T3+T4 (LF-HS + HF-HS), Low vs. high fat: T3 (LF-HS) vs. T4 (HF-HS).

### 3.3 Health scores

All lambs survived to the end of the study (100% survival across treatments). Overall health was good, with most clinical scores close to 1.0 (normal) throughout the experiment (Table 5). Fecal scores, number of days with diarrhea, and diarrhea prevalence did not differ significantly between EWE and any MR treatments. However, fecal scores tended (*P* = 0.07) to be higher in LF-LS lambs than in EWE or high-solids MR lambs. There was no consistent effect of TS concentration (low vs. high) or fat level (low vs. high) on gastrointestinal health indicators. However, lambs on MR had slightly higher cough scores than lambs on EWE (1.06 vs. 1.00; *P* = 0.02). A small but statistically significant difference in ear score was also detected between the low-fat and high-fat MR treatments (*P* = 0.01), although its biological relevance is likely negligible because the numerical difference was minimal (1.00–1.03).

**Table 5:** Health score in lambs (n = 12 per treatment) on natural rearing with ewe milk (EWE) or MR with low fat-low total solids (LF-LS), low fat-high total solids (LF-HS), and high fat-high total solids (HF-HS).

Item	Treatment^a^					*P*-value^a^		
		MR				EWE vs.	Low vs.	Low vs.
	EWE	LF-LS	LF-HS	HF-HS	SEM	MR	high solid	high fat
Diarrhea days	2.10	1.62	1.31	1.03	0.58	0.24	0.52	0.75
Fecal score	1.18	1.22	1.16	1.09	0.04	0.58	0.07	0.27
Ear score	1.00	1.01	1.03	1.00	0.005	0.13	0.70	0.01
Nose score	1.04	1.06	1.05	1.00	0.01	0.80	0.06	0.05
Eye score	1.00	1.00	1.00	1.00	0.001	0.56	0.41	0.16
Cough score	1.00	1.06	1.00	1.06	0.01	0.02	0.10	0.01

a EWE vs. MR: T1 (EWE) vs. T2+T3+T4 (all MR treatments), Low vs. high solid: T2 (LF-LS) vs. T3+T4 (LF-HS + HF-HS), Low vs. high fat: T3 (LF-HS) vs. T4 (HF-HS).

### 3.4 Blood parameters

Serum concentrations of glucose, insulin, thyroxine, and cortisol did not differ among dietary treatments at either sampling time (Table 6). However, notable differences were observed in indices of energy and protein metabolism. On day 20, serum total protein, albumin, and cholesterol were higher in EWE lambs compared with all MR-fed groups. "By weaning (day 63), these differences had largely disappeared for albumin, while serum total protein tended to remain greater in EWE compared with MR-fed lambs (P = 0.07), and EWE lambs maintained higher serum BUN concentrations than MR-fed lambs. Regarding serum BHB, EWE lambs had greater serum BHB concentrations than all MR-fed groups at day 20 (preweaning). By day 63, however, this pattern was reversed, with EWE lambs exhibiting lower BHB concentrations than MR-fed lambs.

**Table 6:** Blood metabolites in lambs (n = 12 per treatment) on natural rearing with ewe milk (EWE) or MR with low fat-low total solids (LF-LS), low fat-high total solids (LF-HS), and high fat-high total solids (HF-HS).

Item	Treatment^a^					*P*-value^a^		
		MR				EWE vs.	Low vs.	Low vs.
	EWE	LF-LS	LF-HS	HF-HS	SEM	MR	high solid	high fat
Glucose, mg/dL								
d20	105	116	111	108	5.60	0.33	0.32	0.68
d63	77.3	68.0	71.2	76.3	2.73	0.10	0.09	0.19
Cholesterol, mg/dL								
d20	103	86.7	81.8	85.2	5.63	<0.01	0.63	0.65
d63	37.1	36.7	41.8	44.3	2.38	0.18	0.03	0.44
BUN, mg/dL								
d20	14.0	14.1	12.4	14.1	0.97	0.68	0.50	0.19
d63	17.0	13.5	13.8	15.6	1.05	0.04	0.34	0.22
BHB, mmol/L								
d20	0.219	0.086	0.088	0.150	0.024	<0.01	0.24	0.06
d63	0.311	0.540	0.434	0.388	0.044	0.01	0.03	0.47
Albumin, g/dL								
d20	4.67	3.96	3.85	4.08	0.12	<0.01	0.95	0.17
d63	4.19	4.13	4.32	4.08	0.094	0.92	0.54	0.07
Total protein, g/dL								
d20	6.16	5.88	5.78	5.76	0.13	0.04	0.48	0.90
d63	5.75	5.61	5.56	5.49	0.087	0.07	0.42	0.55
Insulin, µU/mL								
d20	9.52	12.0	8.66	6.87	2.86	0.92	0.23	0.66
d63	1.01	1.75	2.01	1.24	0.41	0.18	0.85	0.15
Thyroxine, µg/dL								
d20	9.83	9.07	9.42	9.54	0.79	0.61	0.66	0.91
d63	7.55	7.0	6.83	7.86	0.29	0.37	0.33	0.02
Cortisol, µg/dL								
d63	0.655	0.706	0.904	0.784	0.16	0.45	0.48	0.60

a EWE vs. MR: T1 (EWE) vs. T2+T3+T4 (all MR treatments), Low vs. high solid: T2 (LF-LS) vs. T3+T4 (LF-HS + HF-HS), Low vs. high fat: T3 (LF-HS) vs. T4 (HF-HS).

Among MR treatments, LF-LS had lower serum cholesterol and T₄ concentrations than LF-HS and HF-HS lambs at weaning. Although serum glucose did not differ significantly among treatments, LF-LS lambs tended to have lower glucose concentrations than both LF-HS and HF-HS lambs at day 63 (*P* = 0.09).

## 4 Discussion

### 4.1 Rationale for MR nutritional modulation

The Shal sheep is an important Iranian breed characterized by high reproductive performance (approximately 140% lambing rate) and moderate milk yield (about 121 kg over a 143-day lactation period), traits that support its suitability for lamb meat production systems (Tavatori et al., 2005). As reproductive rates increase through genetic approaches such as introgression of the *Booroola* fecundity gene (*FecB*) or nutritional strategies (e.g., flushing), the demand for adequate neonatal nutrition correspondingly intensifies. Under these conditions, maternal milk supply may become insufficient, necessitating the strategic use of MR to meet the nutritional requirements of multiple offspring. Previous research has shown that higher protein concentrations (≥26% CP, DM basis) in MR promote greater ADG and skeletal development when energy supply is adequate (Hill et al., 2008; Zhang et al., 2019), whereas increased fat content elevates dietary energy density and can improve thermoregulation and growth, especially in cold-stressed or low-birth-weight neonates (Ghasemi et al., 2017; Litherland et al., 2014). Similarly, moderate increases in TS achieved through coordinated adjustments in protein and fat have been shown to enhance growth provided that osmolality is properly controlled (Azevedo et al., 2016).

### 4.2 Intake and growth performance

Lambs in the EWE treatment consumed similar or even lower amounts of milk DM (155 g/d) than MR-fed lambs but exhibited the greatest starter intake (234 g/d) during the preweaning period. Although the protein concentration of ewe milk (4.77%) was similar to that of the high-solids MR formulations (4.85%), its considerably greater fat concentration (6.37%vs. 4.27–4.95%) may have promoted earlier satiety during suckling. In addition, the relatively low milk yield per 30-min nursing bout in Shal ewes may have encouraged earlier consumption of solid feed. Similar findings were reported by De la Fuente et al. (1997), who observed lower milk intake but greater starter consumption and more advanced rumen development in naturally suckled than artificially reared lambs. Beyond nutritional factors, the presence of the dam may also stimulate exploratory feeding through social and sensory interactions, thereby promoting starter intake. Consequently, EWE lambs achieved the greatest total ME intake (1.57 Mcal/d), reflecting both the higher energy density of ewe milk and their greater starter consumption. Although direct comparisons among rearing systems are limited, previous studies have consistently reported an inverse relationship between liquid feed allowance and starter intake. For example, increasing MR allowance reduced starter intake in Ghezel lambs, a response that has also been documented in calves offered high volumes of liquid feed (Mohammadi et al., 2024; Shiasi Sardoabi et al., 2021).

Feeding the LF-LS formulation clearly impaired preweaning growth. Although postweaning ADG did not differ among treatments, lambs receiving LF-LS failed to completely compensate for their earlier growth restriction and therefore remained lighter at the end of the experiment. As proposed by Azevedo et al. (2016), the TS concentration of liquid diets is a major determinant of nutrient supply. Because the daily MR allowance was fixed by the feeding protocol, the relatively low nutrient density of the LF-LS formulation (16.3% TS; 0.78 Mcal/kg ME) substantially limited energy and protein intake. Compared with ewe milk, LF-LS contained markedly less fat (3.56%vs. 6.37%) and a lower ME and protein concentration, preventing lambs from consuming sufficient nutrients to support both maintenance and tissue growth. This inadequate nutrient supply likely restricted skeletal development, consistent with previous reports identifying nutrient intake as a major determinant of preweaning growth (Zhang et al., 2019). The reduced growth performance of LF-LS lambs was accompanied by lower serum glucose and greater BHB concentrations at weaning, indicating a more catabolic metabolic state associated with chronic energy deficiency. Because starter intake remained relatively low in this group, the increase in BHB was more likely attributable to mobilization of body fat reserves than to enhanced ruminal ketogenesis. Similar reductions in growth have been reported in lambs fed cow milk–based replacers with lower fat concentrations than ewe milk, emphasizing the importance of matching the nutrient density of maternal milk when formulating MR for artificial rearing (Mousavi Esfiokhi et al., 2024).

In the present study, MR feeding volume was identical among MR treatments (600–1200 mL/d, depending on age), whereas only the TS concentration differed (from 16.3% to 19.6%). Under these conditions, both LF-HS and HF-HS increased milk and starter DM intake compared with LF-LS, resulting in the greatest CP and ME intakes in the EWE and HF-HS groups. These findings differ from studies in which increasing the volume of liquid feed reduced starter intake, a response that has been attributed to delayed rumen development (Zhang et al., 2019). However, because rumen development was not directly assessed in the present study (e.g., by ruminal histology, fermentation characteristics, or microbial colonization), this explanation should be interpreted with caution. Azevedo et al. (2023) similarly concluded that increasing MR TS concentration is a more effective strategy than increasing feeding volume for enhancing nutrient supply without compromising starter intake or nutrient digestibility. Collectively, these results suggest that increasing TS concentration while formulating MR to more closely resemble ewe milk improves nutrient intake without reducing solid feed consumption. Both high-solids formulations contained protein concentrations (4.85%) comparable to ewe milk (4.77%) while providing an appropriate energy supply, thereby maintaining a balanced protein-to-energy ratio. Compared to LF-LS, both LF-HS and HF-HS supported performance comparable with naturally suckled lambs. Relative to LF-LS, both LF-HS and HF-HS supported performance comparable with naturally reared lambs. However, despite consuming more starter DM and ME, HF-HS lambs did not exhibit greater ADG than LF-HS lambs, suggesting that energy supply was no longer the primary constraint once adequate nutrient density had been achieved. Instead, the balance between protein and energy appeared to become a more important role. All treatments provided similar CP:ME ratios (48–52 g CP/Mcal), indicating that when the dietary ratio approaches approximately 50 g CP/Mcal, further increases in energy intake without a proportional increase in protein are unlikely to enhance growth further. This interpretation is consistent with Herath et al. (2020), who reported marked improvements in preweaning growth only when the CP:ME ratio increased substantially (45.9 to 66.5 g/Mcal). Therefore, the absence of a growth advantage in HF-HS compared with LF-HS most likely reflects that the dietary CP:ME ratio was already close to the optimum under the conditions of this study. From a practical perspective, these findings indicate that optimizing the protein-to-energy ratio in MR formulations may be more beneficial than simply increasing dietary fat concentration.

The NRC (2007) does not provide maintenance and growth energy requirements specifically for suckling lambs. Therefore, maintenance ME requirements (MEm) were estimated using the equations for growing lambs (NEm = 0.062 × SBW⁰·⁷⁵; km = 0.644). For LF-LS lambs (average BW = 10.5 kg; ME intake = 1.20 Mcal/d; ADG = 180 g/d), MEm was estimated at 0.56 Mcal/d, leaving 0.64 Mcal/d for growth. Using NRC (2007) values for late-maturing lambs (30% maturity; NEg = 0.42 Mcal/d for 200 g/d; kg = 0.45) predicted an ADG of only 137 g/d, below the observed 180 g/d. This discrepancy suggests that the efficiency of energy utilization for early growth may be greater, or the energy cost of gain lower, than assumed by NRC (2007), possibly because early growth consists predominantly of protein and water accretion rather than fat deposition. In contrast, applying the NASEM (2021) efficiency coefficients (km = 0.69; kg = 0.50), recommended for young animals consuming both milk or MR and starter feed, predicted an ADG of 178 g/d, closely matching the observed value. Similarly, predicted ADG values for EWE, LF-HS, and HF-HS lambs were 217, 221, and 240 g/d, compared with observed values of 221, 224, and 237 g/d, respectively. Thus, NASEM (2021) provided a closer prediction of efficiency coefficients in preweaning growth.

For the postweaning period, the NRC (2007) framework provided much closer predictions. For LF-LS lambs (average BW = 18.5 kg; ME intake = 2.34 Mcal/d; ADG = 264 g/d), estimated ME_m_ was 0.86 Mcal/d, and the calculated total ME requirement was 2.47 Mcal/d, corresponding to a predicted ADG of 248 g/d. For EWE, LF-HS, and HF-HS lambs, predicted ADG values were 308, 285, and 302 g/d, compared with observed values of 306, 281, and 298 g/d, respectively. Overall, NRC (2007) predicted postweaning growth with deviations of less than 10 g/d across treatments, indicating good agreement between predicted and observed performance during this period.

Skeletal measurements provide reliable indicators of nutritional status and long-term growth potential. Lambs fed LF-LS exhibited reduced hip and withers heights at weaning (day 63), and these differences persisted through day 83, reflecting their lower nutrient intake. These results are consistent with previous studies in calves fed high-solids MR (Shiasi Sardoabi et al., 2021) or fat-supplemented diets (Ghasemi et al., 2017). In contrast, HF-HS lambs exhibited greater skeletal development, particularly in withers height (day 63) and hip width (day 83), suggesting that the combination of higher TS (19.6%) and fat concentration (4.85%) supported skeletal growth at least as effectively as natural suckling. This pattern aligns with observations by De la Fuente et al. (1997), who reported greater carcass fat deposition in naturally suckled lambs, likely due to higher milk fat intake. In our study, the enhanced skeletal growth in HF-HS lambs, despite lower milk fat than ewe milk, may reflect a more favorable protein-to-energy ratio (∼51 g CP/Mcal), promoting lean tissue deposition and skeletal growth rather than fat accretion. The greater hip width in HF-HS lambs compared with LF-HS lambs suggests that the additional fat and CP intake may have been partitioned toward structural development. This interpretation is further supported by the higher serum thyroxine (T4) concentrations observed in HF-HS lambs at weaning, because thyroid hormones play an important role in skeletal growth and metabolic regulation. However, because growth hormone and insulin-like growth factor-1 (IGF-1) were not measured, the underlying endocrine mechanisms remain speculative.

### 4.3 Health and metabolism

Preweaning mortality remains a major challenge in sheep production systems worldwide, with reported mortality rates ranging from 5 to 30% (Gascoigne et al., 2023). In the present study, however, survival was 100% from day 3 to day 103 regardless of treatment, consistent with the findings of Huang et al. (2023). This outcome was most likely attributable to appropriate management practices, including adequate colostrum intake, hygienic housing conditions, and the use of high-quality MR.

Neonatal diarrhea is a multifactorial disorder influenced by nutritional, environmental, and immunological factors. Among dietary factors, osmolality plays an important role in maintaining gastrointestinal health. In calves, MR osmolality exceeding 500 mOsm/kg has been associated with an increased risk of abomasal bloat, whereas values above 600 mOsm/kg may impair intestinal barrier function (Azevedo et al., 2023). As expected, increasing the TS concentration increased MR osmolality in the present study. However, partial replacement of lactose with fat in the HF-HS formulation limited this increase, resulting in a lower osmolality than that of the LF-HS formulation (466 vs. 506 mOsm/kg). Despite these differences, neither treatment adversely affected health status or diarrhea incidence. This observation may reflect the relatively slow rate of milk consumption through nipple bottles, which could reduce the osmotic load imposed on the abomasum.

Although fecal scores remained within the normal range (1.0–1.2) in all treatments, LF-LS lambs showed numerically higher fecal scores and experienced more days with diarrhea than lambs receiving the high-solids formulations, despite the lower osmolality and sugar concentration of the LF-LS diet. This apparently contradictory finding suggests that the greater nutrient density and energy supply provided by the high-solids MR formulations may have promoted gastrointestinal health, possibly through better energy status and intestinal homeostasis. However, because markers of immune function (e.g., immunoglobulins and cytokines) were not evaluated, the underlying mechanisms remain speculative (Amdi et al., 2021; Jasper and Weary, 2002). These observations are consistent with the findings of Shiasi Sardoabi et al. (2021), who demonstrated that moderately increasing MR total solids (12 to 17%), while maintaining an appropriate balance of protein and fat, improved gastrointestinal health in calves. Respiratory health was also unaffected by treatment. Although MR-fed lambs had marginally greater cough scores than ewe-reared lambs (1.06 vs. 1.00), this difference was biologically negligible and was not associated with any other clinical signs of respiratory disease. Likewise, ear, eye, and nasal scores remained within the normal range throughout the experiment, indicating none of the MR formulations adversely affected the health status of the lambs.

Blood metabolites provided further insight into the metabolic adaptation of lambs during the preweaning period. The lower serum cholesterol concentration observed in LF-LS lambs at weaning, compared with the high-solids MR groups, likely reflected their lower energy and fat intake. In growing ruminants, serum cholesterol is primarily derived from hepatic synthesis and dietary fat absorption (NRC, 2007). The LF-LS diet, with the lowest fat content (3.56%vs. 4.27–4.95% in HF-HS and LF-HS) and ME concentration (0.78 vs. 0.93–0.95 Mcal/kg), provided less substrate for hepatic very-low-density lipoprotein synthesis and reduced dietary cholesterol supply. Consequently, the lower serum cholesterol concentration observed in LF-LS lambs was consistent with their lower ME intake and poorer growth performance. This interpretation is further supported by the observation that cholesterol concentrations in HF-HS lambs were comparable to, or even exceeded, those in EWE lambs by day 63, suggesting that the fat-enriched, high-solids MR provided sufficient lipid substrate to maintain normal cholesterol metabolism.

Although serum glucose did not differ significantly among treatments, the tendency for lower glucose concentrations in LF-LS lambs compared with LF-HS and HF-HS lambs deserves consideration. The LF-LS diet provided less total sugars (7.55%vs. 9.04% and 8.32% in LF-HS and HF-HS, respectively) and lower ME intake, which may have reduced glucose precursor availability. Moreover, the elevated BHB concentrations in LF-LS lambs at weaning suggest that these animals relied more heavily on lipid mobilization to meet their energy requirements.

Serum BHB a marked treatment × age interaction, reflecting two distinct physiological processes. On day 20, ewe-reared lambs had the greatest serum BHB concentrations, possibly because of their greater starter intake. In young ruminants, butyrate produced during ruminal fermentation is converted to BHB within the ruminal epithelium; therefore, under adequate nutritional conditions, elevated serum BHB is generally considered an indirect indicator of rumen development rather than metabolic stress (Suarez-Mena et al., 2017). However, because ruminal morphology, fermentation characteristics, and microbial colonization were not evaluated, conclusions regarding rumen development should be interpreted cautiously. This interpretation agrees with the findings of De la Fuente et al. (1997), who reported greater rumen development in naturally suckled lambs than in artificially reared lambs based on direct measurements. Lambs receiving the LF-LS treatment exhibited the greatest serum BHB concentrations despite having the lowest starter intake and metabolizable energy intake. Under these conditions, the elevated BHB concentrations most likely reflected increased mobilization of body fat reserves in response to inadequate energy intake rather than enhanced ruminal fermentation (Rivas et al., 2020). These findings emphasize that the physiological interpretation of serum BHB depends on nutritional context. Under adequate nutrient supply, elevated BHB may indicate greater ruminal fermentation activity, whereas under energy restriction it is more likely to reflect increased lipid mobilization and catabolic metabolism. In contrast, HF-HS lambs exhibited lower BHB concentrations than LF-HS lambs at weaning, suggesting a more favorable energy balance resulting from their greater nutrient intake. Serum thyroxine (T4) concentrations declined progressively from day 20 to weaning, consistent with the normal developmental pattern reported in growing lambs. Nevertheless, T4 concentrations at weaning were greatest in HF-HS lambs, exceeding those of LF-LS lambs and remaining comparable to those of EWE group. This finding suggests that the high-solids, fat-enriched MR formulation maintained metabolic activity at a level comparable with natural suckling. Conversely, the lower T4 concentration observed in LF-LS lambs, together with their elevated serum BHB concentrations, supports the conclusion that these lambs experienced a less favorable energy status during the preweaning period. Serum albumin, an indicator of protein nutritional status and hepatic synthetic capacity, total protein and BUN concentrations were greater in EWE than in all MR-fed groups. This difference may reflect differences in either protein intake or protein composition between ewe milk and the MR formulations. However, as direct measurements of amino acid profiles, or protein quality were not directly evaluated, the nutritional mechanisms responsible for this difference cannot be determined from the present study.

### 4.4 Limitations

Several limitations of the present study should be considered when interpreting the findings. First, the EWE treatment differed from the MR treatments in both diet and rearing system (natural suckling vs. artificial feeding). Although housing, starter feeding, and general management were standardized across treatments, the absence of a bottle-fed ewe milk group prevented complete separation of the effects of milk composition from those of maternal contact. Nevertheless, the primary objective of this study was to evaluate whether practical MR formulations could achieve growth and health outcomes comparable to those obtained under conventional natural suckling. Accordingly, ewe-reared lambs served as the biologically relevant reference group. Second, rumen development was evaluated indirectly through starter intake and serum BHB concentrations rather than by direct measurements of ruminal fermentation, morphology, histology, or microbial colonization. Consequently, conclusions regarding rumen development should be interpreted with appropriate caution. Third, although the sample size (n = 12 per treatment) was sufficient to detect differences in growth performance, it may have limited the statistical power to identify relatively small differences in health-related variables. Fourth, estimation of milk intake in ewe-reared lambs using the weigh–suckle–weigh technique is subject to inherent measurement error, and the controlled suckling schedule may not fully represent natural nursing behavior. Fifth, this trial was conducted using a single fat-tailed sheep breed (Shal), which may restrict the direct generalizability of the findings to other breeds or differing commercial farm management systems. Moreover, although individual lambs (n = 12 per treatment) provided sufficient statistical power for body weight, skeletal growth, and blood parameters, the number of pen replicates (n = 3 per treatment) was relatively limited for pen-level variables for starter feed intake. Finally, because multiple clinical, health, and metabolic endpoints were evaluated simultaneously without universal family-wise statistical adjustments, there is an inherent risk of false-positive findings (Type I error) for secondary outcomes displaying minor or marginal significance.

## 5 Conclusion

Feeding the LF-LS (16.3% TS) MR resulted in inferior preweaning growth and a lower serum cholesterol, a tendency toward lower glucose, and higher BHB concentrations at weaning, suggesting a less favorable energy status. In contrast, increasing TS to 19.6% (LF-HS and HF-HS) improved nutrient intake, skeletal growth, and preweaning ADG to levels comparable to those of naturally suckled lambs. Compared with LF-HS, HF-HS increased energy intake and hip width, but did not improve ADG, suggesting that increasing energy density beyond this level did not further enhance growth. Despite the higher osmolality of the high-solids MR formulations, fecal scores and the number of diarrhea days remained similar among treatments, indicating that osmolality values of up to approximately 500 mOsm/kg were well tolerated and did not adversely affect the gastrointestinal health indicators evaluated in this study. Overall, these findings demonstrate that a high-solids MR formulated to closely match the ME and CP composition of ewe milk and approximately 50 g CP/Mcal ME can support growth performance comparable to natural suckling in Shal lambs. Nevertheless, these findings should be confirmed in other sheep breeds, production systems, and over longer production periods.

## Ethics statement

All experimental procedures involving animals were reviewed and approved by the Institutional Animal Care and Use Committee (IACUC) of Isfahan University of Technology, Isfahan, Iran. The study was conducted in strict accordance with national guidelines for the care and use of experimental animals under the supervision of the Research Vice-Chancellorship of Isfahan University of Technology and in collaboration with Isfahan University of Medical Sciences. The experiment was carried out at the Lavark Educational–Research Sheep Farm, College of Agriculture, Isfahan University of Technology, Isfahan, Iran. Additional information on the institutional ethics policy (available in Persian) is accessible at: https://research.iut.ac.ir/fa/dgr/3043.

## Ethics declaration

This study was conducted in accordance with the ARRIVE (Animal Research: Reporting of In Vivo Experiments) guidelines. This study was approved by the Institutional Animal Care and Use Committee (IACUC) of Isfahan University of Technology, Isfahan, Iran.

## CRediT authorship contribution statement

**Ameneh Akbarizadeh:** Writing – original draft, Resources, Methodology, Investigation, Formal analysis, Data curation. **Ebrahim Ghasemi:** Writing – review & editing, Writing – original draft, Visualization, Validation, Supervision, Software, Project administration, Investigation, Funding acquisition, Formal analysis, Data curation, Conceptualization. **Meysam Gholami:** Resources, Methodology, Investigation, Data curation. **Bahareh Dolatkhah:** Visualization, Supervision, Resources, Project administration, Methodology, Investigation, Funding acquisition, Conceptualization.

## Declaration of competing interest

The authors declare the following financial interests/personal relationships which may be considered as potential competing interests: Novin Roshdeh Shahran Fodeh Company provided the milk replacer and technical assistance with milk formulation. The company had no role in the design of the study, the collection, analysis, or interpretation of data, the writing of the manuscript, or the decision to submit the manuscript for publication. All other authors declare no competing interests.
